# The research focus and frontiers in surgical treatment of essential tremor

**DOI:** 10.3389/fneur.2024.1499652

**Published:** 2024-12-11

**Authors:** Linlin Zhang, Shifang Cui, Hongyan Bi, Qiang Chen, Mengfan Kan, Cheng Wang, Yu Pu, Hongxia Cheng, Bin Huang

**Affiliations:** ^1^Nantong Fourth People's Hospital, Nantong, China; ^2^Heilongjiang University of Chinese Medicine, Harbin, China; ^3^Shandong University of Traditional Chinese Medicine, Jinan, China

**Keywords:** essential tremor, surgery, hotspots, DBS, MRgFUS

## Abstract

**Background:**

Essential tremor (ET) is one of the most prevalent neurodegenerative disorders, with surgery serving as the principal treatment option. This paper presents a bibliometric analysis of research in the field of ET surgery from 2004 to 2024, aiming to identify current research hotspots and inform future research directions.

**Methods:**

This study employs CiteSpace to analyze publication trends, countries/institutions, authors, keywords, and co-cited references in ET surgery, using the Web of Science core database from 2004 to 2024 to delineate the research pathways.

**Results:**

A total of 1,362 publications were included in this study. The number of publications has shown steady growth over the analyzed period from 2004 to 2024. Research in this field was carried out in 58 countries and by 371 institutions. The United States had the highest volume of publications, with the University of California System identified as the most prolific institution. Dr. Michael S. Okun from the University of Florida was the most prolific author, also based in the United States. This study identified 879 keywords, with significant citation bursts noted in areas such as the caudal zona incerta, ventral intermediate nucleus, location, and MR-guided focused ultrasound. Among the top ten highly cited articles, five pertained to MR-guided focused ultrasound thalamotomy, two addressed localization techniques, and one focused on surgical targets.

**Conclusion:**

This study employs comprehensive bibliometric and visualization analyses to elucidate the evolution of research and identify emerging hotspots. The identified hotspots are as follows: First, deep brain stimulation (DBS), the most advanced technology in ET surgery, has room for improvement, especially in neuromodulation automation. Second, MR-guided focused ultrasound thalamotomy is a new surgical approach that requires further research on efficacy, safety, and side effect management. Third, novel surgical targets have demonstrated some efficacy, yet further research is essential to validate their effectiveness and safety. Lastly, localization techniques are fundamental to ET surgery, with ongoing efforts directed towards achieving more precise, individualized, and intelligent localization.

## 1 Introduction

Essential tremor (ET) is the most common movement disorder in adults, leading to significant disability, interfering with daily activities, and diminishing quality of life (1). The global prevalence of ET is approximately 0.9%, reaching a peak of 5% in individuals aged 65 and older (2). Although pharmacological treatment is the primary therapeutic approach for ET, many patients exhibit poor responses to medication or suffer from severe side effects (3). For those with drug-resistant ET, surgical intervention presents the most promising option.

The generation of tremors involves a network comprising the cerebellum, thalamus, and motor cortex, interconnected through structures such as the Guillain-Mollaret triangle, the anterior thalamic tract, and the cortical-cerebellar pathways (4, 5). Lesions in any component of this network can lead to a reduction in tremor manifestation. Surgical intervention targets this network’s structure to alleviate tremors.

ET surgery can be classified into neuromodulation and thalamotomy based on the method of intervention. Neuromodulation includes deep brain stimulation (DBS) and spinal cord stimulation. DBS targeting the ventral intermediate nucleus (VIM) is currently the most established method in the field of ET surgery, with compelling evidence supporting the efficacy and safety of bilateral DBS (6, 7). Spinal cord stimulation modulates neural signals by implanting electrodes around the spinal cord and providing electrical stimulation; however, research in this area is currently limited (8, 9). Thalamotomy encompasses magnetic resonance-guided focused ultrasound (MRgFUS) and thermal ablation (10). MRgFUS, as an emerging minimally invasive procedure method, offers significant advantages for patients seeking to avoid the risks and invasiveness of surgery. However, thermal ablation techniques remain irreplaceable in certain situations, such as for patients with low skull density ratio (SDR). Furthermore, the exploration of new targets may offer better options for ET patients (11). Progress in targeting and localization techniques complements each other, as more precise localization provides technological support for the discovery and validation of new therapeutic targets (12–14).

In recent years, the field of ET surgery has garnered significant interest. However, a comprehensive exploration and analysis of research in this area are still lacking. Bibliometric analysis, through quantitative methods and visualization techniques, can objectively reveal the research hotspots and trends in the field of ET surgery. Compared to traditional review methods, this analytical approach provides a more comprehensive and objective description of the development trends in ET surgery, strengthening the rationality of the research method and offering more precise scientific evidence for clinical decision-making (15). This study employs bibliometric analysis to visualize relevant literature published over the past two decades in the field of ET surgery, utilizing the Web of Science core database. By conducting co-occurrence analysis on countries, institutions, authors, keywords, and highly-cited documents in this field, it uncovers research hotspots and trends, identifies deficiencies and areas for improvement in current research, and aids clinicians in understanding the latest treatment technologies and their development dynamics, thus providing robust support for clinical decision-making and enhancing its practicality for practitioners.

## 2 Materials and methods

Literature pertaining to essential tremor was obtained from the Web of Science core database (Science Citation Index Expanded (SCIE)). The search strategy employed was: (essential tremor) AND (surgery OR surgical OR stimulation OR radiofrequency ablation OR MRI-guided laser interstitial thermal therapy OR MR-guided focused ultrasound OR stereotactic radiotherapy OR Gamma knife thalamotomy OR cell transplantation therapy OR gene therapy). The inclusion criteria were as follows: (1) publication dates from 2004 to August 2024; (2) no restrictions on language; (3) limited to literature type: Articles. A total of 1,362 articles were retrieved. Pure text files were selected to export the “full records” and “citation references.” Following the extraction, the data obtained from WoSCC was downloaded and imported into CiteSpace software, where duplicates were removed, maintaining a final count of 1,362 articles.

**Figure 1:**
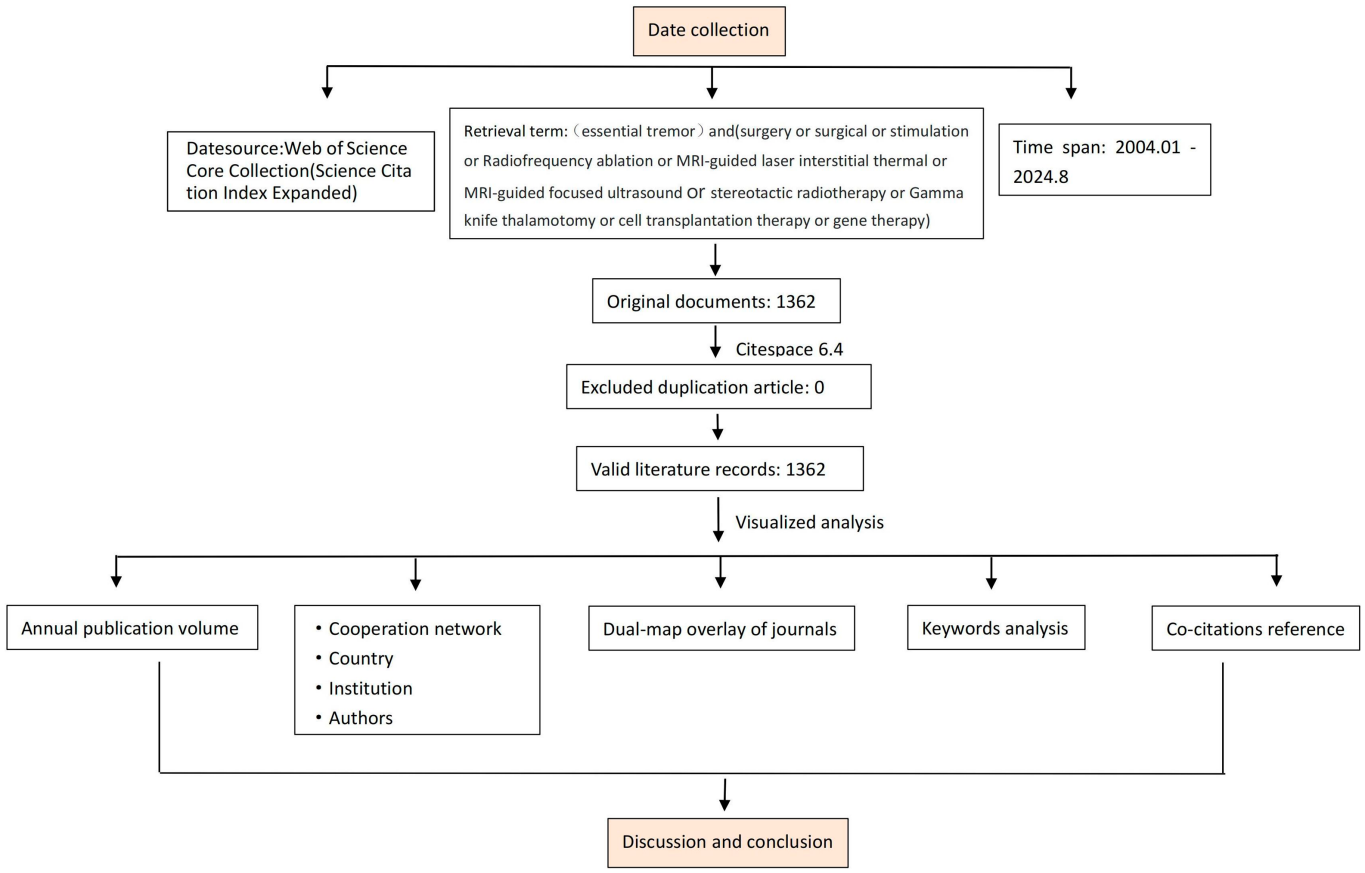
The flow chart of the study design.

CiteSpace is a software specifically designed for the analysis of scientific literature. It is primarily based on the concept of “co-occurrence clustering,” which involves extracting information units from scientific literature (such as references, keywords, authors, institutions, etc.) and reconstructing the network structure based on the type and strength of connections between these information units. The CiteSpace parameters included a one-year time slice, duration from January 2004 to August 2024, term source: all items, one node type at a time. All other settings were retained at their default values (Figure 1).

## 3 Results

### 3.1 Publication trends

**Figure 2:**
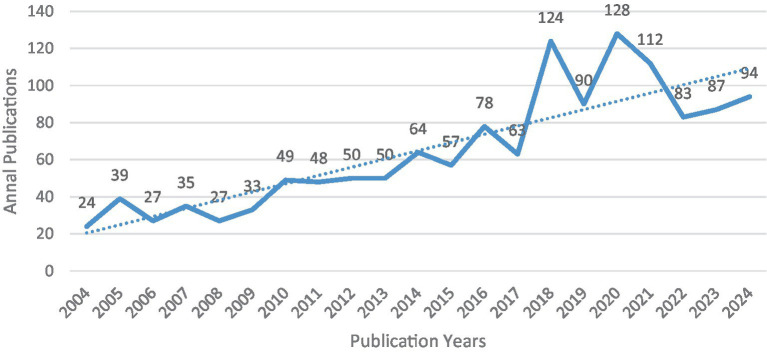
The annual number of global publications and citations.

In recent years, the number of publications focused on surgical interventions for essential tremor has steadily increased (Figure 2). This trend can be categorized into three phases: the first phase, from 2004 to 2016, displayed steady growth in publication volume; the second phase, from 2017 to 2020, saw an explosive surge in publications; and the third phase, from 2021 to the present, has resulted in a near stabilization in publication volume.

### 3.2 Analysis by country or region

Over the past decade, researchers from 58 countries have conducted studies on surgical interventions for essential tremor. Among these, the USA leads with 664 publications, accounting for one-third of the total output and establishing itself as a central research country in this field. Following the USA are Germany (205), England (110), Canada (110), and France (72), which comprise the top five countries in terms of publication volume.

**Figure 3:**
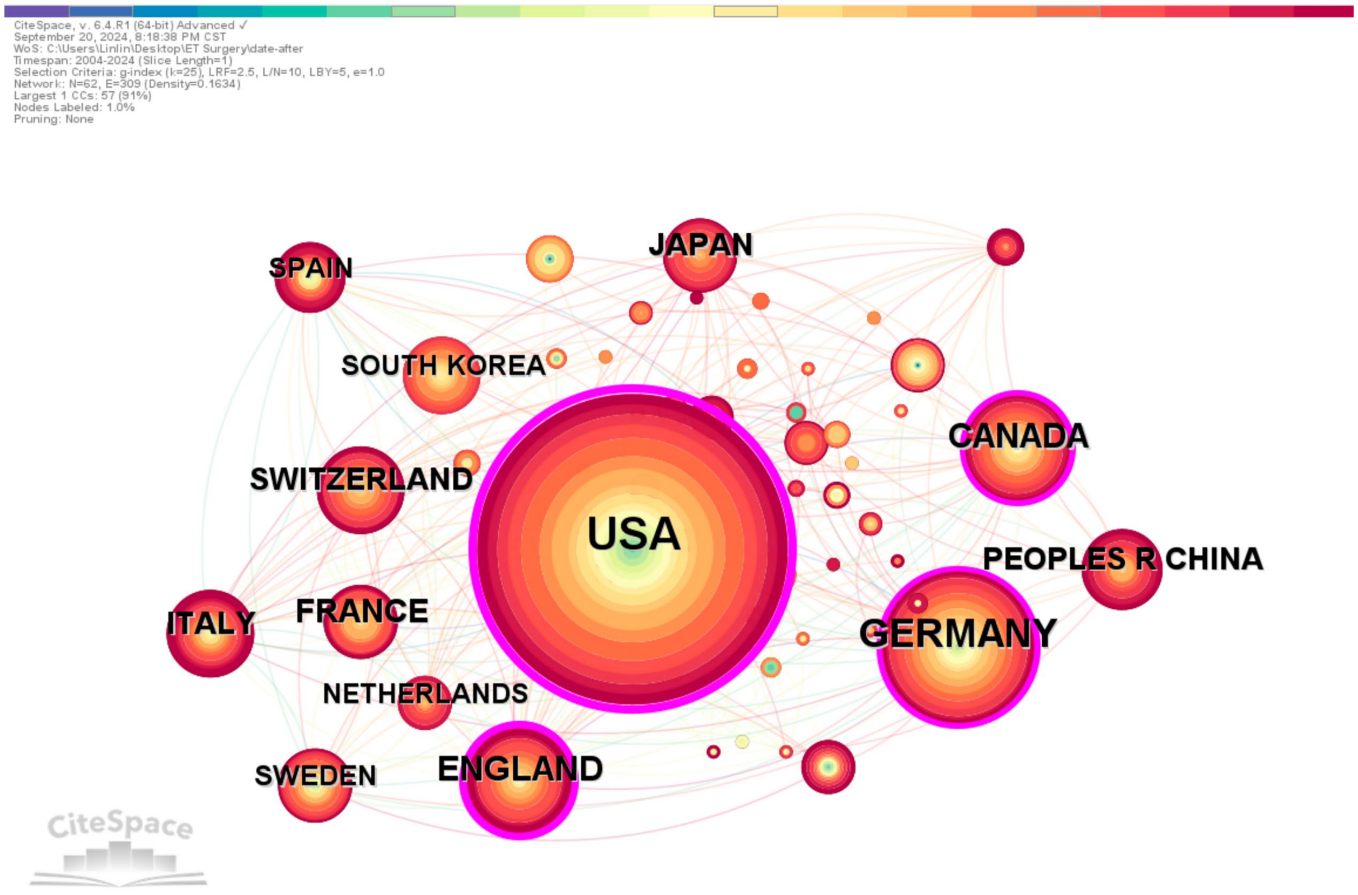
Country co-occurrence. The colored bar at the top represents the years, with the years increasing from left to right. The publication volume, divided by country/institution, is indicated by the size of the circles. The lines separating the circles represent international collaborations between countries/institutions. The more connections, the closer the cooperation with other countries/institutions. The purple outer ring represents the betweenness centrality, and the thicker the purple outer ring, the greater the betweenness centrality.

In the CiteSpace visualization, each node represents a country, with nodes featuring a purple ring indicating high centrality (centrality ≥0.1), signifying their importance and influence. The USA exhibits the highest centrality (0.6), indicating its significant impact within this field. In terms of international collaboration, countries work closely together, particularly the USA, Germany, and Canada, which collaborate with all other nations ranked in the top 10 for publication volume (Figure 3; Table 1).

**Table 1:** The top 10 institutes and countries in terms of publication count and centrality.

Rank	Country	Count	Centrality	Institution	Count	Centrality
1	USA	664	0.6	University of California System	87	0.11
2	Germany	205	0.14	University of Toronto	80	0.02
3	England	110	0.28	State University System of Florida	64	0.07
4	Canada	110	0.19	University Health Network Toronto	58	0.06
5	France	72	0.08	University of Florida	58	0.02
6	Japan	67	0.03	Harvard University	54	0.08
7	Switzerland	64	0.06	Mayo Clinic	48	0.06
8	Italy	62	0.05	Helmholtz Association	40	0.02
9	Peoples R China	58	0.01	University of Cologne	40	0.01
10	Sweden	53	0.03	Centre National de la Recherche Scientifique (CNRS)	39	0.02

**Figure 4:**
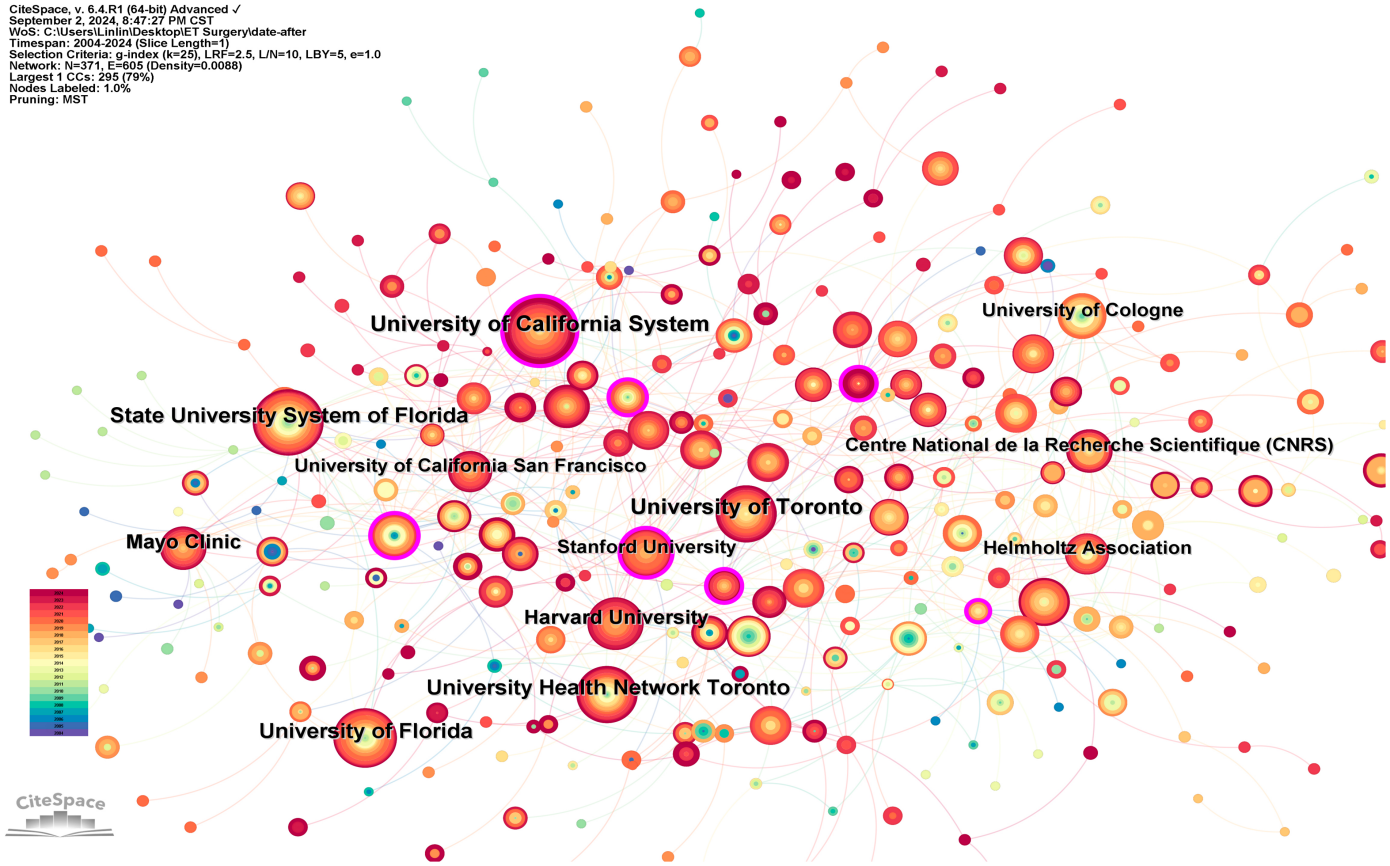
Research collaboration map between institutions. The color bar in the lower left corner represents the years, and the deeper the red color of the nodes in the figure, the more recent the year.

Among the top ten institutions by publication volume, five are based in the United States. The institution with the most publications is the University of California System (87), followed by the University of Toronto (80), the State University System of Florida (64), the University Health Network Toronto (58), and the University of Florida (58). However, the collaborative relationships between institutions are complex, with extensive partnerships particularly noted between institutions in the USA and Canada. Figure 4 illustrates that the University of California and Stanford University have the largest purple circles, indicating their central positions in institutional collaborations. However, the cooperation among institutions shows a geographical bias, with institutions in the United States having more connections with each other and fewer links with institutions in other countries.

### 3.3 Co-authorship analysis

**Figure 5:**
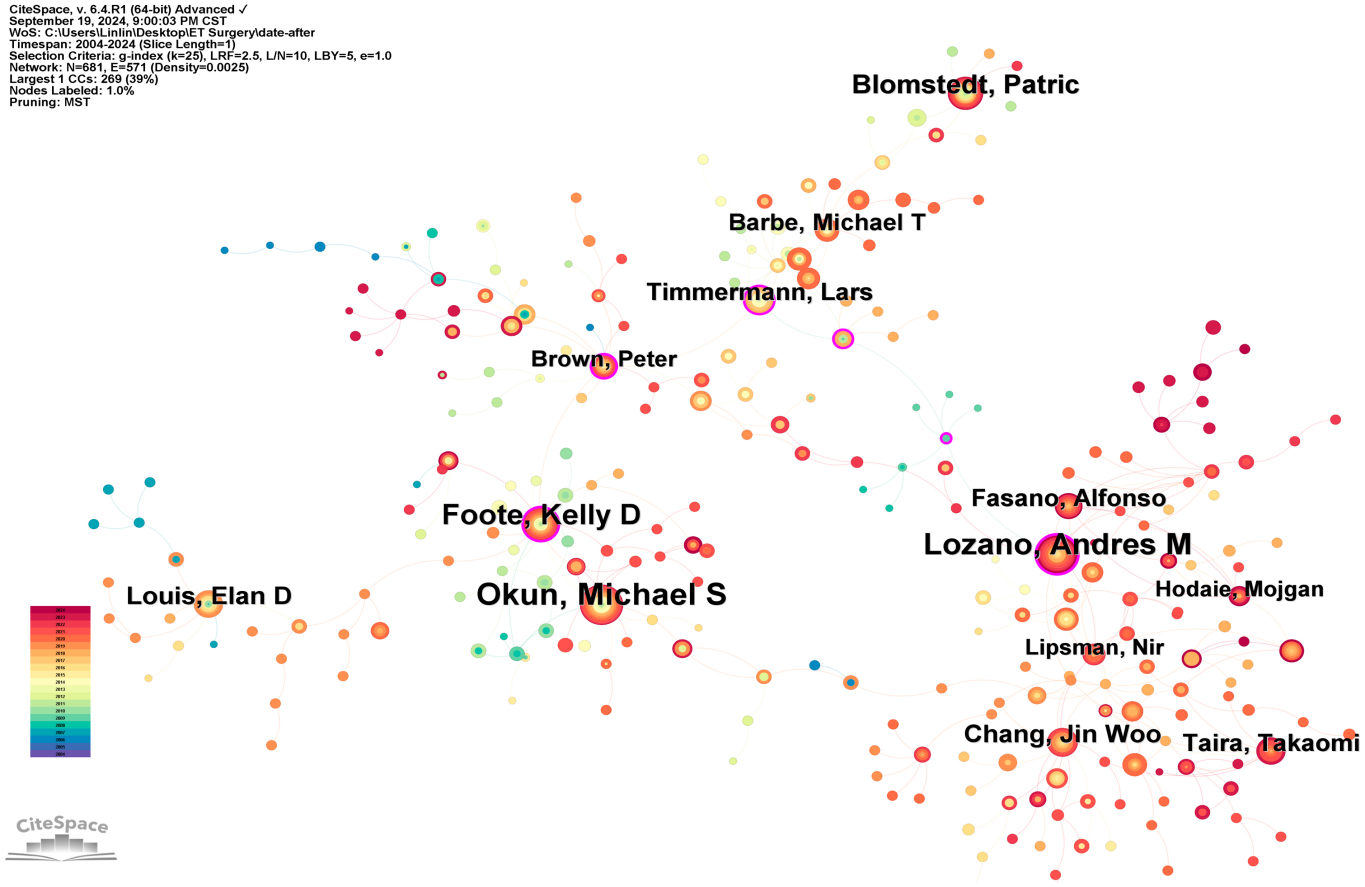
Author co-occurrence. The size of the circles represents the number of articles published by the authors, with the circles representing the authors themselves. The lines between the circles represent collaborations between authors. The more intense the collaboration with other authors, the more lines there are.

The co-authorship analysis shows that a total of 681 authors have contributed to the field of ET surgery (Figure 5; Table 2). The author with the highest publication volume is Michael S. Okun from the University of Florida, who has published 43 papers in this area. However, he primarily collaborates with Kelly D. Foote, a fellow researcher at the same institution, resulting in a relatively low centrality score (0.03). According to the co-authorship network, Andres M. Lozano occupies a central position, exhibiting a centrality score of 0.15. He collaborates not only with Alfonso Fasano from his institution but also with Takaomi Taira from Tokyo Women’s Medical University and Jin Woo Chang from Yonsei University. In contrast to Lozano, the collaboration patterns among the other authors exhibit regional characteristics, leading to the formation of three small research groups. In addition, Figure 5 shows that authors are divided into three research communities characterized by geography, centered around Lozano, Andres M, Okun, Michael S, and Timmerman, Lars.

**Table 2:** The top 10 authors and their organizations.

Rank	Author	Institute, Country	Count	Centrality
1	Okun, Michael S	Univ Florida	43	0.03
2	Lozano, Andres M	University of Toronto	40	0.15
3	Foote, Kelly D	Univ Florida	33	0.12
4	Blomstedt, Patric	Umea Univ	23	0.01
5	Chang, Jin Woo	Yonsei University	20	0.04
6	Louis, Elan D	Univ Texas Southwestern	19	0.03
7	Taira, Takaomi	Tokyo Womens Med Univ	18	0.02
8	Fasano, Alfonso	Univ Toronto	18	0.01
9	Timmermann, Lars	University of Cologne	18	0.14
10	Barbe, Michael T	University of Cologne	15	0.04

### 3.4 Keyword analysis

#### 3.4.1 Keyword co-occurrence analysis

**Figure 6:**
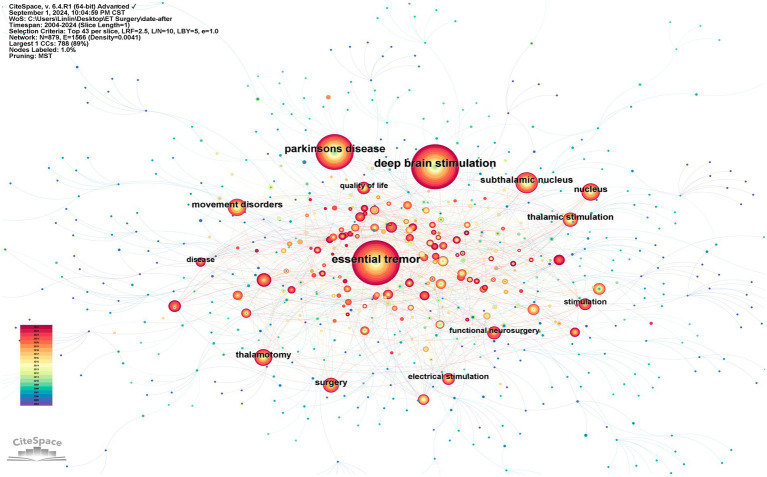
A network map of co-occurring keywords. Circles represent keywords, with the size of the circles indicating the frequency of keyword occurrence. The lines between nodes indicate that keywords appear together.

The keyword co-occurrence analysis reflects the primary research focuses within this field. Using citation frequency as the primary metric for observation, the ten most frequently cited keywords, excluding subject terms, are: “deep brain stimulation,” “subthalamic nucleus,” “movement disorders,” “surgery,” “thalamic stimulation,” “nucleus,” “thalamotomy,” “electrical stimulation,” “stimulation,” and “disease” (Figure 6; Table 3).

**Table 3:** The top 10 most frequent keywords.

Rank	Keywords	Count	Centrality
1	Deep brain stimulation	779	0.02
2	Subthalamic nucleus	234	0.05
3	Movement disorders	175	0.06
4	Surgery	151	0.08
5	Thalamic stimulation	148	0.08
6	Nucleus	140	0.05
7	Thalamotomy	135	0.05
8	Electrical stimulation	108	0.04
9	Stimulation	108	0.06
10	Disease	83	0.12

#### 3.4.2 Keyword clustering

**Figure 7:**
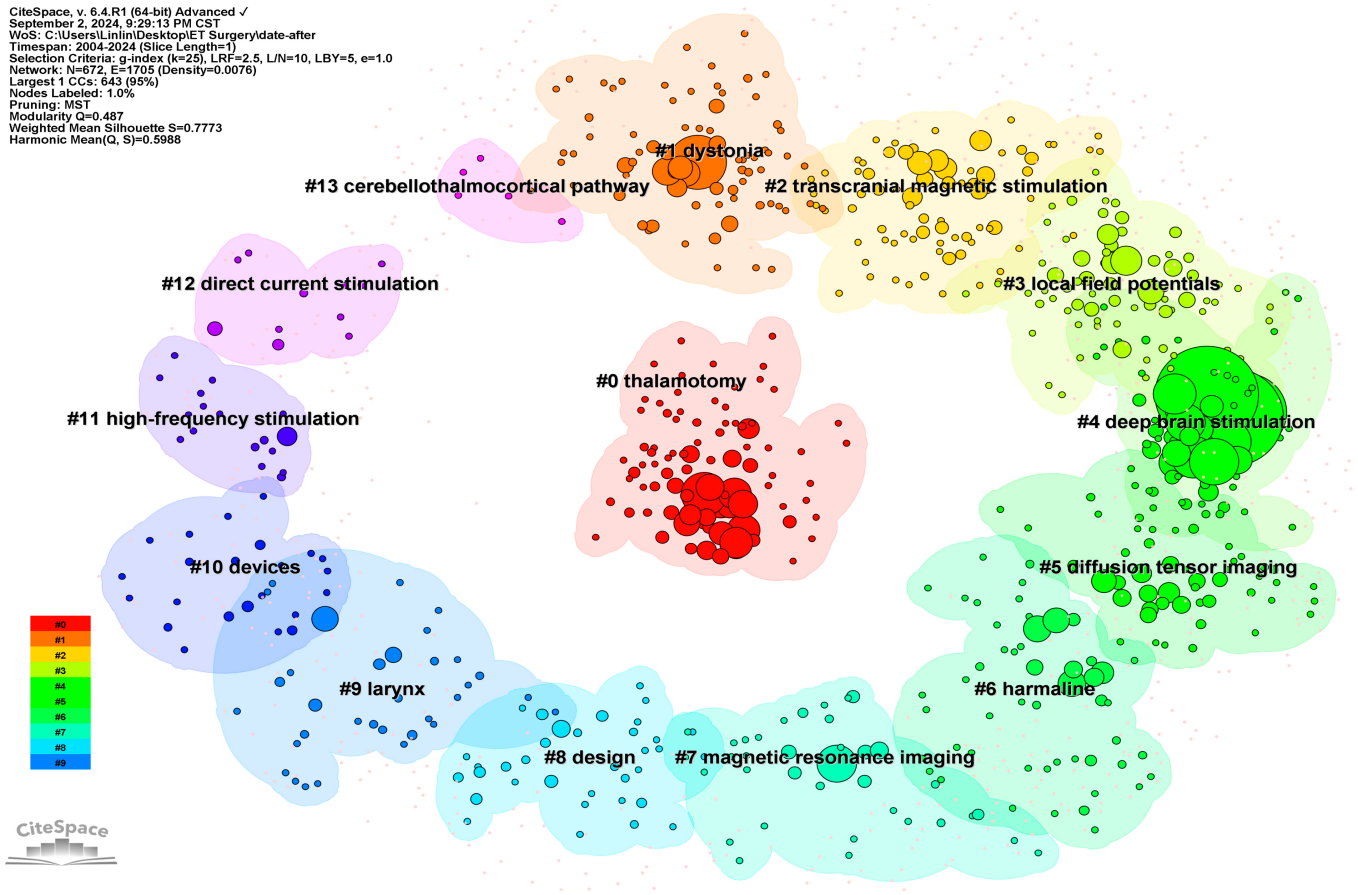
Clustering map of co-occurring keywords. The colored areas in the figure represent clusters, and the nodes represent the keywords within the clusters.

The clustering diagram of co-occurring keywords visually illustrates the interconnections and thematic groupings of these research areas. The Q and S values in the clustering diagram serve as measures of result reliability; a Q value greater than 0.3 and an S value greater than 0.7 indicate a reliable clustering result. For this analysis, the Q value for keyword clustering is 0.487, and the S value is 0.7773, demonstrating the reliability of these clustering outcomes. This analysis successfully identified 14 distinct cluster labels: #0 thalamotomy, #1 dystonia, #2 transcranial magnetic stimulation, #3 local field potentials, #4 deep brain stimulation, #5 diffusion tensor imaging, #6 harma line, #7 magnetic resonance imaging, #8 design, #9 larynx, #10 devices, #11 high-frequency stimulation, #12 direct current stimulation, and #13 cerebello-thalamocortical pathway (Figure 7).

#### 3.4.3 Keyword burst analysis

**Figure 8:**
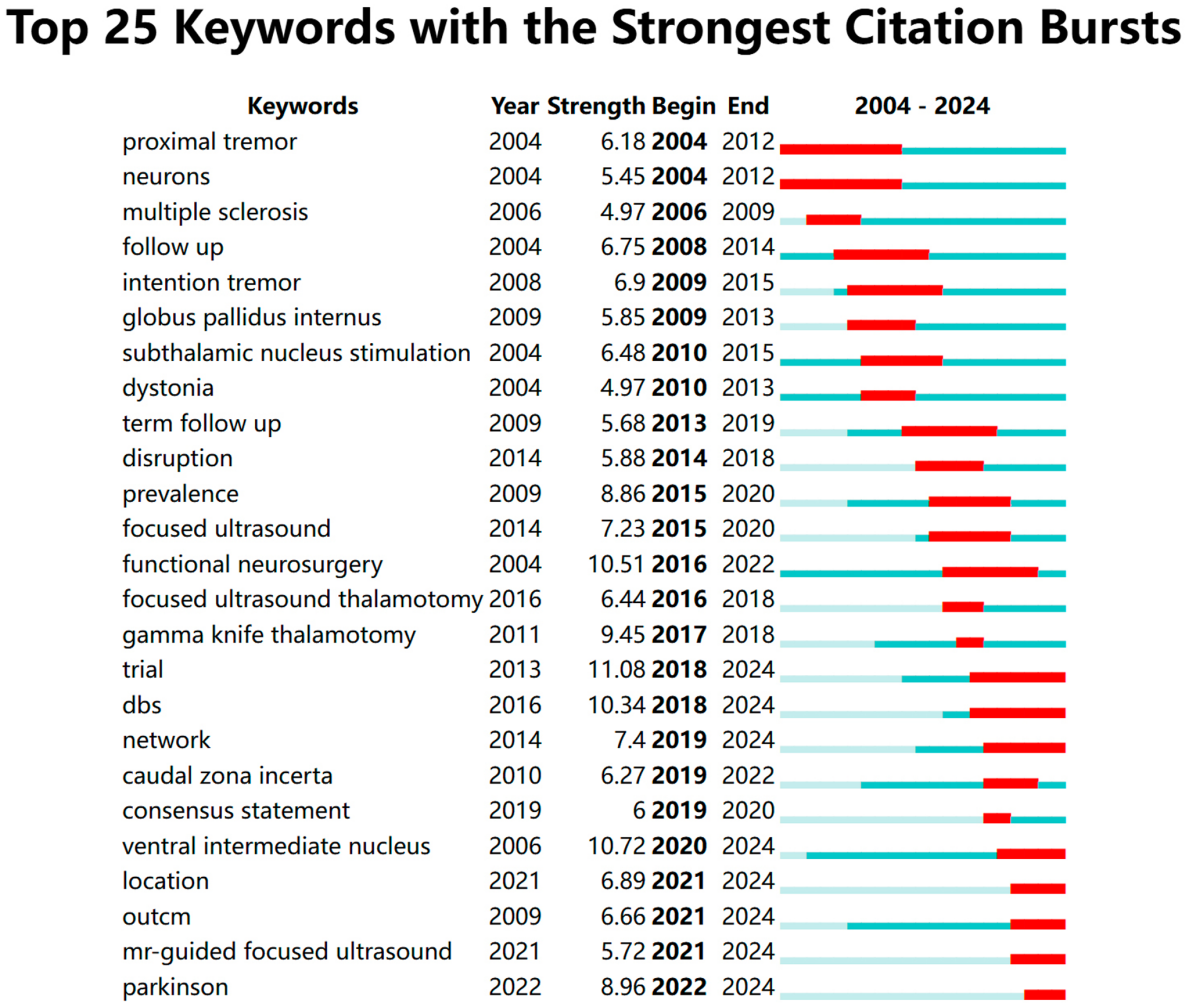
The top keywords with the strongest citation bursts. Keyword burst analysis was employed. In the keyword burst analysis, “start” and “end” indicate the time of the burst. “Intensity” refers to the strength of the burst, representing the reliability over time.

The burst analysis of keywords clearly reflects the research hotspots in this field over specific time periods (Figure 8). This analysis can be divided into three phases: the first phase, from 2004 to 2012, identified hotspot keywords primarily focused on “proximal tremor, neurons, multiple sclerosis, follow up, intention tremor, globus pallidus internus, subthalamic nucleus stimulation, dystonia.” This phase primarily discussed the improvement of tremor symptoms due to surgical interventions. The second phase, from 2013 to 2018, recognized hotspot keywords that included “term follow up, disruption, prevalence, focused ultrasound, functional neurosurgery, focused ultrasound thalamotomy, gamma knife thalamotomy.” During this period, focused ultrasound thalamotomy and gamma knife thalamotomy began to gain prominence. The third phase, from 2018 to the present, comprises keywords such as “trial, DBS, network, caudal zona incerta, consensus statement, ventral intermediate nucleus, location, outcome, MR-guided focused ultrasound, Parkinson.” Both deep brain stimulation and magnetic resonance-guided focused ultrasound remain predominant research areas, while the selection of surgical location is attracting increasing attention from researchers.

### 3.5 Co-citation analysis

**Figure 9:**
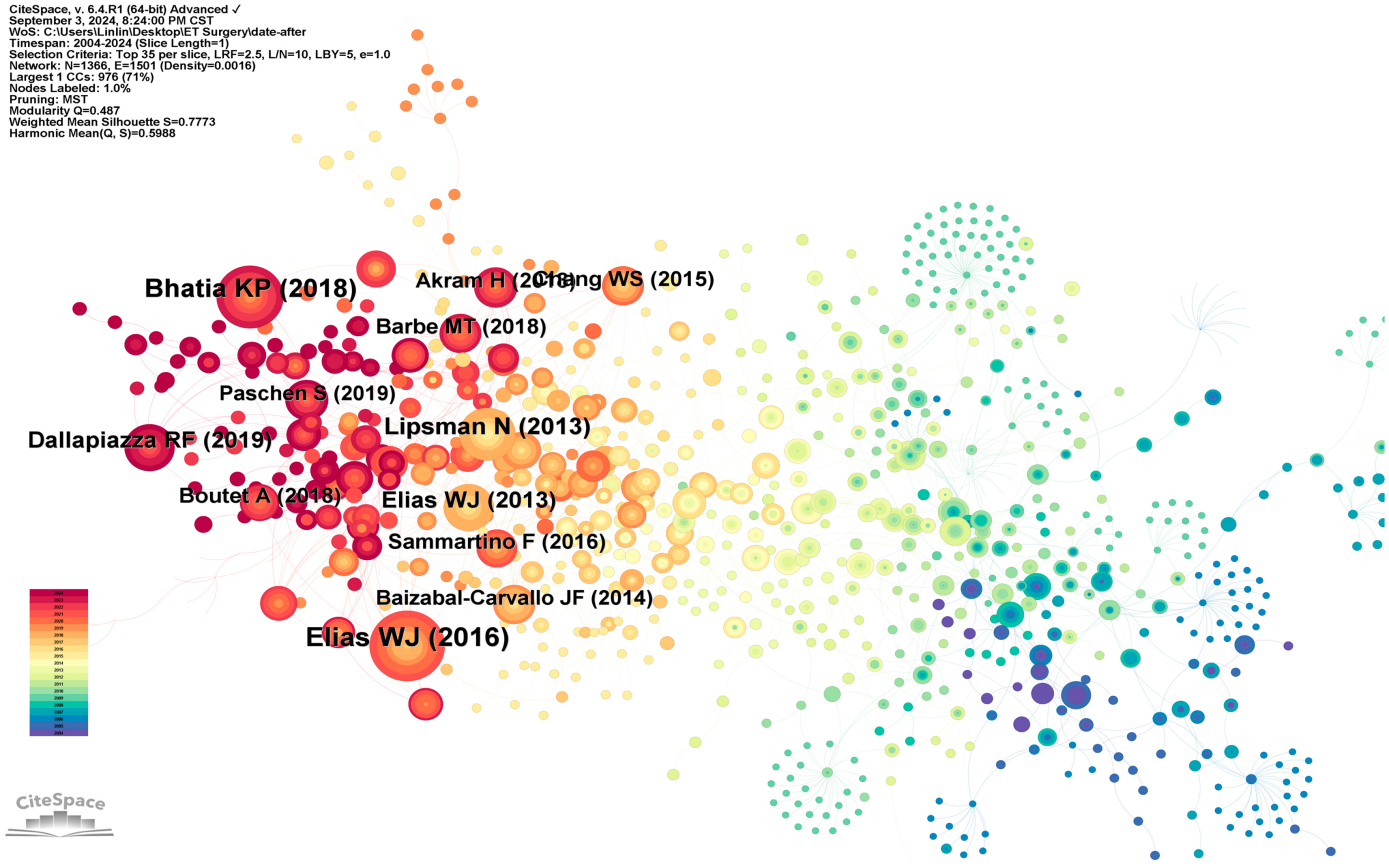
Highly-cited publications and co-cited references. In the figure, nodes represent documents. The color of the nodes indicates the publication time of the documents, with darker blue signifying older publications and darker red indicating more recent ones.

Co-citation occurs when two or more papers are cited by another paper, establishing a connection between the cited literature and the co-cited literature. A visual map generated using “references” as nodes reveals a total of 1,366 nodes (Figure 9). The ten most frequently cited documents include three randomized controlled trials (RCTs) (11, 16, 17), three before-after studies (18–20), one non-controlled trial (16), two reviews (21, 22), and one cross-sectional study (23). In these documents, there are five articles on focused ultrasound thalamotomy, two studies explored the relationship between surgical target location and clinical benefits, and one validated the feasibility of new localization techniques to improve the accuracy of VIM nucleus targeting (Table 4).

**Table 4:** The top 10 highly cited studies.

Rank	Cited reference	Title	Co-citation	Centrality	Type	Year
1	Elias WJ, 2016, NEW ENGL J MED, V375, P730, DOI 10.1056/NEJMoa1600159	A Randomized Trial of Focused Ultrasound Thalamotomy for Essential Tremor	116	0.02	RCT	2016
2	Bhatia KP, 2018, MOVEMENT DISORD, V33, P75, DOI 10.1002/mds.27121	Consensus Statement on the classification of tremors. From the task force on tremor of the International Parkinson and Movement Disorder Society	95	0	Review	2018
3	Lipsman N, 2013, LANCET NEUROL, V12, P462, DOI 10.1016/S1474-4422(13)70048-6	MR-guided focused ultrasound thalamotomy for essential tremor: a proof-of-concept study	66	0.05	BA study	2013
4	Elias WJ, 2013, NEW ENGL J MED, V369, P640, DOI 10.1056/NEJMoa1300962	A pilot study of focused ultrasound thalamotomy for essential tremor	56	0.01	NCT	2013
5	Dallapiazza RF, 2019, J NEUROL NEUROSUR PS, V90, P474, DOI 10.1136/jnnp-2018-318240	Outcomes from stereotactic surgery for essential tremor	53	0.02	Review	2019
6	Chang WS, 2015, J NEUROL NEUROSUR PS, V86, P257, DOI 10.1136/jnnp-2014-307642	Unilateral magnetic resonance guided focused ultrasound thalamotomy for essential tremor: practices and clinicoradiological outcomes	41	0.02	BA study	2015
7	Akram H, 2018, NEUROIMAGE-CLIN, V18, P130, DOI 10.1016/j.nicl.2018.01.008	Connectivity derived thalamic segmentation in deep brain stimulation for tremor	41	0.01	BA study	2018
8	Barbe MT, 2018, NEUROLOGY, V91, PE543, DOI 10.1212/WNL.0000000000005956	DBS of the PSA and the VIM in essential tremor: A randomized, double-blind, crossover trial	40	0.01	RCT	2018
9	Sammartino F, 2016, MOVEMENT DISORD, V31, P1217, DOI 10.1002/mds.26633	Tractography-Based Ventral Intermediate Nucleus Targeting: Novel Methodology and Intraoperative Validation	38	0.08	RCT	2016
10	Boutet A, 2018, BRAIN, V141, P3405, DOI 10.1093/brain/awy278	Focused ultrasound thalamotomy location determines clinical benefits in patients with essential tremor	38	0.03	Cross-Sectional	2018

## 4 Discussion

### 4.1 Current research status

In recent years, the volume of literature concerning essential tremor has experienced significant growth, with the most rapid increase occurring between 2017 and 2019. Institutions in the United States, particularly the University of California system, have published the majority of papers in this field and exert substantial influence. There is considerable collaboration among various countries and institutions. Michael S. Okun from the University of Florida has authored the highest number of papers, with his research primarily focusing on surgical target selection and localization techniques.

### 4.2 Research hotspots and trends

Keyword co-occurrence, bursts, and bibliometric analyses reveal shifting research hotspots within this field. Recent years have seen deep brain stimulation (DBS), MR-guided focused ultrasound thalamotomy, surgical positioning, and localization techniques emerge as the primary research foci in surgical treatments for essential tremor.

#### 4.2.1 Deep brain stimulation

DBS involves delivering electrical impulses to specific nuclei in the brain (e.g., VIM or PSA) through a drilled hole in the skull while the patient is under local anesthesia, aiming to regulate abnormal neural signals to suppress or alleviate tremors (24). Since its FDA approval in 1997, DBS has played a crucial role in tremor treatment. Unlike radiofrequency ablation or gamma knife radiosurgery (25), DBS does not destroy brain tissue, thereby minimizing the risk of permanent damage. The effects of DBS are immediate, with therapeutic outcomes observable shortly after surgery.

However, DBS is not a permanent solution; parameters must be continuously adjusted in response to disease progression and symptom changes (26). Consequently, automation has been introduced in this area. Researchers (27–29) have implemented real-time detection of neural signals to automatically adjust stimulation intensity and other parameters based on patient needs. An innovative fully implanted bidirectional neural interface can detect neural activity during stimulation and includes circuits for feedback control (29). However, this technology is not yet fully developed, and our understanding of various brain signals remains limited—this understanding is a prerequisite for automated adjustments (30). Additionally, algorithms for adjusting stimulation frequency still pose a challenge, necessitating further research for optimization (31).

#### 4.2.2 MR-guided focused ultrasound thalamotomy

Recently, MR-guided focused ultrasound thalamotomy has emerged as an important research focus in essential tremor. Among the ten most cited papers in this area, six pertain to MR-guided focused ultrasound thalamotomy. The study “A Randomized Trial of Focused Ultrasound Thalamotomy for Essential Tremor” by Elias et al. (16), which garnered the highest number of citations, details the enrollment of 76 patients with essential tremor (ET) across eight international research centers. These patients were randomly assigned in a 3:1 ratio to undergo either MRgFUS or a sham procedure. In the MRgFUS group, the hand tremor score improved from a baseline of 18.1 to 9.6 at 3 months, whereas the sham group’s score changed marginally from 16.0 to 15.8. The improvements in the MRgFUS group were maintained at the 12-month mark.

MRgFUS thalamotomy is a minimally invasive procedure that can be performed while the patient is awake, thereby reducing surgical risks (32). The therapeutic effects of MRgFUS thalamotomy are reversible, allowing for repeat treatments or adjustments to the treatment area if necessary. This technique presents broader indications, offering an alternative treatment option for patients unable to accept the risks associated with DBS, those with concerns about implanted devices, or individuals who are unsuitable candidates for DBS (12, 33).

However, MRgFUS thalamotomy also has its limitations. Bone density and structure may interfere with ultrasound transmission, and patients with intracranial metal are not suitable for MRI scans. Furthermore, caution is warranted in patients with cognitive or psychiatric disorders (12, 33). On the other hand, MRgFUS thalamotomy improved hand tremor scores by 47% at 3 months, with this improvement lasting for 12 months (16). Bilateral VIM DBS can reduce overall tremors by 66–78%, with improvement rates of 60.3–75% for upper limb action tremors 5 years post-surgery. Consequently, the efficacy of MRgFUS thalamotomy is somewhat inferior to that of DBS, and there is a lack of long-term efficacy follow-up studies on MRgFUS thalamotomy. Large-sample, multicenter, and long-term evaluation studies are needed.

#### 4.2.3 Surgical targets

DBS targeting the ventral intermediate nucleus (VIM) is a well-established, effective, and safe treatment method (34). However, recent studies have identified the posterior subthalamic area (PSA) (35)and caudal zona incerta (cZI) (36, 37) as potentially more advantageous stereotactic targets. In the highly-cited literature, two articles discussed the clinical benefits of different targets. Barbe et al. (11) found PSA-DBS to be more effective than VIM-DBS, producing optimal stimulation effects at lower amplitudes and providing at least equivalent tremor control, with no differences in the quality or quantity of side effects between the two targets. However, side effects associated with PSA-VIM stimulation were more prominent regarding emotional and behavioral disturbances, likely due to PSA’s involvement in emotional regulation (38). Another relevant study conducted a double-blind crossover trial comparing VIM DBS with PSA DBS (39), noting a wiring trajectory that crossed both regions, with a dorsal contact point in VIM, a neutral contact point on the intercommissural line (ICL), and a ventral contact point in PSA. Follow-up after 12 months revealed no statistically significant difference in tremor suppression between VIM and PSA, although there was a slight trend toward better tremor suppression with PSA stimulation.

Some researchers propose that the efficacy of PSA and VIM DBS in essential tremor may depend on the distance to the dentate nucleus. Dembek et al. (35) conducted a randomized crossover trial involving 13 essential tremor patients implanted with contacts in both VIM and PSA. Three months post-implantation, they calculated the Euclidean distance from the electrode contacts in PSA and VIM to the center of the dentate nucleus using patient-specific MRI images aligned with a population-averaged DRTT model. The results demonstrated that the PSA contacts were closer to the dentate nucleus, resulting in superior tremor suppression on TRS scores.

cZI is another alternative target for ET DBS. The zona incerta is an area located beneath the thalamus that functions in the regulation of movement and pain transmission. Stimulation of cZI is believed to influence the entire motor network, including circuits from the cerebellum and basal ganglia, potentially improving tremor symptoms through modulation of movement-related neurotransmission (36). Eisinger et al. (40) conducted DBS on 47 essential tremor patients and found that the short-term benefits (after 6 months and 2 years) of VIM and cZI were similar. However, long-term follow-up (3–4 years) revealed a decreasing trend in tremor scores over time for VIM DBS, whereas cZI DBS exhibited an increasing trend; thus, cZI DBS appeared less effective in long-term tremor control compared to VIM DBS. Moreover, cZI DBS may lead to side effects related to cerebellar and motor control, such as unsteadiness or motor incoordination (41), underscoring the need for effective monitoring and management strategies to mitigate these adverse effects. Furthermore, the surgical procedure for cZI DBS is more challenging and complex due to its location beneath the thalamus and proximity to other critical neural structures, making precise target localization a significant challenge.

Additionally, the efficacy of MRgFUS may also depend on the location of the target. Arcadi et al. (12) found that the effectiveness and acute adverse reactions of MRgFUS largely rely on the precise location and size of the focused ultrasound thalamotomy.

#### 4.2.4 Localization techniques

Accurate targeting of the surgical site is critical for successful outcomes in both DBS and MRgFUS thalamotomy. There is a variety of localization techniques, including ventriculography, stereotactic frames, and MRI guidance. However, as research on targets becomes more in-depth, more complex targets (such as PSA and cZI) require more precise localization to determine their positions.

Target structures such as PSA and cZI are complex and challenging to localize, necessitating advancements in localization technology for the application of new DBS targets. Given current developmental and practical needs, specific breakthrough directions are warranted. Firstly, due to individual anatomical variations in brain structures, a universal stereotactic frame may not precisely accommodate each patient’s unique anatomy. Therefore, utilizing patient-specific MRI data to create personalized brain models can enhance localization accuracy. Secondly, postoperative brain shift is a significant concern; the brain tissue may shift following surgery, potentially disrupting the alignment between preoperative imaging and actual brain tissue (42). Thus, the development of imaging techniques capable of providing real-time feedback is essential. Lastly, artificial intelligence has become a burgeoning area of interest. Through advanced image processing techniques, pattern recognition, and machine learning algorithms, AI can accurately analyze and identify brain structures, optimize fiber tracking, compensate for real-time brain tissue displacement, and personalize surgical planning, significantly improving localization precision in deep brain stimulation surgeries.

## 5 Conclusion

This study employs comprehensive and systematic bibliometrics to visually analyze the literature on the surgical treatment of essential tremor (ET) over the past 20 years, identifying research hotspots in the field of ET surgery. This provides valuable directions for future clinical practice and research in this area, and also enables readers to gain a comprehensive understanding of peer education, training, and research guidance.

The findings of this research identify the following hotspots: 1. In terms of technology, DBS is currently the most established technique, offering significant advantages in safety and side effect management; current research focuses on automating neuroregulation via DBS. 2. MRgFUS thalamotomy, as an emerging method, is gaining attention but has limitations regarding safety and side effect management. 3. There are still many areas to explore regarding surgical target selection, including validating the efficacy of emerging targets, managing side effects, and assessing patient indications. 4. Localization techniques are foundational to surgery, and precise localization is essential for surgical success. For challenging-to-localize targets like PSA and cZI, accurate, safe, and rapid localization techniques will help facilitate breakthroughs in target selection research. This study elucidates the aforementioned research hotspots and frontier areas, aiming to provide researchers and clinical practitioners in the field with a clearer direction for development and to offer support and guidance for future research.

## Data Availability

The original contributions presented in the study are included in the article/supplementary material, further inquiries can be directed to the corresponding author.
